# As Social Media Scales Back Fact-Checking, Can Technologies Fill the Gap?

**DOI:** 10.2196/95730

**Published:** 2026-04-06

**Authors:** Wendy Glauser

**Keywords:** misinformation, social media, artificial intelligence, fact-checking, algorithms, public health

**Key Takeaways**

- Algorithms, artificial intelligence fact-checking, and social media ads can reduce the spread of misinformation at scale.
- Low-cost “content-neutral” interventions reminding users to think before sharing can help prevent misinformation spread.

*Part one of this series* [1] *showed how researchers are working with social media influencers to boost accurate health information online. In part two, we explore technological solutions for detecting and combating misinformation*.

Misinformation is increasingly spread with single clicks, bots, and artificial intelligence (AI) deepfakes. AI-generated images and videos share fake treatments, with even deepfake versions of renowned doctors’ likenesses used to gain credibility [2]. In an age where generative AI is increasing the volume and speed of health misinformation [3] and agencies like the World Health Organization are raising alarms about the impact on vaccine trust and public health [4], are AI and algorithm-based technologies for combating that misinformation keeping up?

While evidence suggests technological solutions to misinformation on social media are effective, researchers worry that social media companies’ interest in employing, evaluating, and improving these tools has waned in recent years.

Common technologies for combating misinformation include everything from algorithmic labeling of posts that contain misinformation, to downregulation of AI-deemed inaccurate posts, to mass awareness campaigns that encourage critical thinking [5-7].

Cameron Martel, PhD—assistant professor of marketing at the Johns Hopkins Carey Business School—explains that in the late 2010s and early 2020s, major social media companies, including Facebook and Twitter, employed algorithms to identify potentially false articles and engaged third-party fact-checkers to verify posts.

In 2023, he led a large study of warning labels, in which over 14,000 participants in the United States were exposed to true and false headlines and asked about their belief in the headlines or interest in sharing them [8]. Half of the participants were exposed to warning labels when presented with false information, while half were not.

Fact-checking labels reduced belief in false information by nearly 28% and reduced misinformation sharing by roughly 25% relative to the control group. The study also showed that among those with low trust in fact-checkers, warning labels nonetheless reduced misinformation sharing by more than 16%.

In January 2025, however, Meta announced it would end its partnership with third-party fact-checkers and instead adopt community notes, whereby everyday users comment on the accuracy of information [9]. If comments are upvoted by people from across the political spectrum, then they’ll appear prominently.

Such community notes are likely to be trusted if the process behind community note generation is transparent and reasonable, Martel says. In a study published last year, Martel and colleagues [10] found that while both Democrat- and Republican-leaning participants preferred expert fact-checkers over laypeople, laypeople “juries” could be deemed equally trustworthy as or more trustworthy than experts if their size was large enough, they had consulted with each other, and they had equal representation across political groups.

## The Rise of AI Fact-Checking

There is far less information about how the public views AI fact-checking tools and their accuracy. A study (available as a preprint [11]) suggests that the large language models (LLMs) Perplexity and Grok largely align with community note decisions about posts that are misleading. However, 21% to 28% of posts that community notes deemed as misleading were deemed true by the AI bots.

Concerningly, the authors observe that the launch of the Grok bot on X in early March 2025 co-occurred with a substantial reduction in the community note submissions, suggesting that social media users may see AI as an alternative, rather than as a complement, to democratized fact-checks.

While Martel points out that AI can be very helpful for identifying and responding to “well debunked conspiracy theories or often repeated myths,” the limit of AI fact-checking has become glaring during breaking news events. Al Jazeera reported, for example, that Grok struggled to recognize AI-generated media in conflict situations and incorrectly said that a trans pilot was responsible for a helicopter crash, among many other breaking news fact-checking errors [12].

“Large language models don’t have any existing corpus of information about what’s happening currently,” explains Martel. Yet, “there’s at least anecdotal evidence that people are still trying to use LLMs to find out information about unfolding events, and that is troubling.”

Martel says that ultimately, democratized fact-checking through community notes, AI fact-checking, and professional fact-checkers “have great promise” when used in tandem. For example, AI systems could refer breaking news or claims that they can’t easily verify to human fact-checkers, social media users could rate the accuracy of information fact-checked by AI, and AI and algorithm-based systems could respond to real-time feedback from democratized fact-checks.

But fact-checking systems should be transparent, continually audited, assessed for effectiveness, and improved. And that’s not happening. “Right now, it seems like there is no corporate will to invest heavily in these types of content moderation practices, so while I’m theoretically hopeful about these technologies, in practice, I’m less hopeful,” says Martel.

## “Content-Neutral” Interventions Can Promote Critical Thinking on Social Media

Interventions that are “content neutral” are another scalable solution to reducing misinformation, says Hause Lin, PhD—a researcher at the Massachusetts Institute of Technology and Cornell University, and a data scientist at the World Bank. “People are going to be producing all kinds of weird content that you just will not be able to anticipate,” he explains, but interventions that encourage critical thinking and help people spot common propaganda tactics can blunt the influence of misinformation.

In 2023, Lin and colleagues [7] assessed the effectiveness of Facebook and Twitter ads that encouraged people to consider the accuracy of information before they shared it. The Facebook study, which involved 33 million users, found that these accuracy prompts led to a 2.6% reduction in misinformation sharing among users who had previously shared misinformation (as flagged by third-party fact-checkers or Facebook’s internal system). The Twitter study, which relied on data from over 157,000 users, showed that accuracy prompts resulted in an up to 6.3% reduction in misinformation sharing among users who saw at least one ad and had previously shared misinformation.

The magnitude of the effect could be much higher with different types of accuracy prompts that are designed to reach more people over longer periods of time, Lin says. (The Facebook study only assessed user behavior for an hour after the ad was shared, while the Twitter study evaluated user behavior over days to weeks, Lin explains.) Regardless, 6% of millions is a significant impact for a relatively “low-cost” intervention.

The goal of the project was to jolt social media users from an emotional state to a reflective state, Lin says. “When people are scrolling, they are often not thinking reflectively but intuitively. They’re thinking ‘This gets me worked up so I’m going to share it with the world,’” he explains. “If you slow them down just a little bit, and say, ‘Do you want to think more about whether this is true?’ that actually reduces misinformation.”

Still, Lin acknowledges that large-scale content moderation may not align with the profit motive. For example, Lin recently studied the effect of “prosocial” celebrity messages aimed at countering ethnic hate–driven rhetoric on social media in Nigeria. A preprint of the study [13] suggests that people who saw the videos were less likely to share hate content but also more likely to reduce the time they spent on Twitter overall. “The side effects of interventions like this can be unpredictable,” Lin says.

There is growing evidence that multipronged efforts can help counter health and other misinformation, and even small efforts can make an impact. Whether social media companies are willing to invest in these initiatives for the broader social good remains to be seen.
